# High efficiency of benzoporphyrin derivative in the photodynamic therapy of pigmented malignant melanoma

**DOI:** 10.1038/sj.bjc.6690131

**Published:** 1999-02

**Authors:** A Busetti, M Soncin, G Jori, M A J Rodgers

**Affiliations:** Department of Biology, University of Padova, Padova, Italy; Center for Photochemical Sciences, Bowling Green State University, Bowling Green, OH, USA

**Keywords:** photodynamic therapy, pigmented melanoma, benzoporphyrin derivative

## Abstract

Benzoporphyrin derivative monoacid ring A (verteporfin, BPD-MA) when intravenously injected (5.5 μmol kg^−1^) to C57/BL6 mice bearing a subcutaneously transplanted B1 melanoma gave a maximal accumulation in the tumour within 1–3 h with recoveries of 1.84– 1.96 μmol kg^−1^. Irradiation of BPD-MA-loaded melanoma with 690-nm light from a dye laser at 3 h and 9 h post injection induced tumour necrosis and delay of tumour growth of 28 and 14 days respectively. The response of the tumour to BPD-MA photosensitization was enhanced by pretreatment with 1064-nm light from a pulse-operated Nd:YAG laser, which caused a selective breakdown of melanosomes. © 1999 Cancer Research Campaign


*British Journal of Cancer* (1999) **79**(5/6), 821–824
© 1999 Cancer Research Campaign
Article no. bjoc.1998.0131

### High efficiency of benzoporphyrin derivative in the photodynamic therapy of pigmented malignant melanoma

**A Busetti[1]*, M Soncin[1]*, G Jori[1] and MAJ Rodgers[2]**

[1]Department of Biology, University of Padova, Italy; [2]Center for Photochemical Sciences, Bowling Green State University, Bowling Green, OH, USA

**Summary** Benzoporphyrin derivative monoacid ring A (verteporfin, BPD-MA) when intravenously injected (5.5 μmol $kg^{-1}$) to C57/BL6 mice bearing a subcutaneously transplanted B1 melanoma gave a maximal accumulation in the tumour within 1–3 h with recoveries of 1.84–1.96 μmol $kg^{-1}$. Irradiation of BPD-MA-loaded melanoma with 690-nm light from a dye laser at 3 h and 9 h post injection induced tumour necrosis and delay of tumour growth of 28 and 14 days respectively. The response of the tumour to BPD-MA photosensitization was enhanced by pretreatment with 1064-nm light from a pulse-operated Nd:YAG laser, which caused a selective breakdown of melanosomes.

**Keywords:** photodynamic therapy; pigmented melanoma; benzoporphyrin derivative

Photodynamic therapy (PDT) mediated by Photofrin has recently been approved as a therapeutic modality for specific types of malignant tumours (Levy, 1996). This breakthrough further stimulates research efforts aimed at developing more effective phototherapeutic protocols, as well as at broadening the scope of PDT and overcoming its present limitations. Thus, it is well known (Dougherty, 1987) that PDT with Photofrin is essentially ineffective in the treatment of heavily pigmented tumours, largely owing to the strong absorbance of melanin in the 630-nm range, i.e. at the wavelengths that are most frequently used to activate Photofrin in clinical PDT; as a consequence, melanin inhibits any significant light penetration into this type of neoplastic lesions (Svaasand et al, 1990). Recent findings (Schuitmaker et al, 1995; Biolo et al, 1996; Woodburn et al, 1997) show that an experimentally implanted melanotic melanoma can be made to be responsive to PDT when the irradiation is performed in the presence of selected second-generation photosensitizing agents, such as Si(IV)-naphthalocyanine, bacteriochlorin a and Lu(III)-texaphyrin; all of these photosensitizers are characterized by an expanded macrocycle and high molar absorptivity in the 750–800 nm spectral interval, where melanin exhibits a small residual absorbance, thereby minimizing its optical filtering action (Svaasand et al, 1990). The efficacy of the PDT treatment is further enhanced if the melanosomes in the tumour are extensively destroyed by preirradiation with high peak power pulsed laser radiation at 1064 nm (Busetti et al, 1998).

At present, the studies with these photosensitizers are progressing in order to optimize their phototherapeutic activity. On the other hand, we found that a promising newly developed PDT agent, benzoporphyrin derivative monoacid ring A (verteporfin, BPD-MA), displays particularly large photosensitizing efficacy against B16 pigmented melanoma, i.e. a heavily pigmented variety of melanotic melanoma, in spite of the fact that its longest wavelength absorption band peaks at 690 nm, where the transmittance of pigmented mammalian tissues to light is still very low. This favourable but unexpected property of BPD-MA is the subject of the present communication.

### MATERIALS AND METHODS

Female C57/BL6 mice (18–20 g body weight) obtained from Harlan Sprague Dawley (Indianapolis, IN, USA) were used as experimental models. B16 pigmented melanoma (the B16 cell line was obtained from American Type Culture Collection) was subcutaneously transplanted into the upper flank of the mice by injecting 20 μl ($10^6$ cells) of a sterile cell suspension in phosphate-buffered saline (PBS). The cell line was cultured in Dulbecco's modified minimal essential medium (Sigma) and supplemented with 10% fetal calf serum (Boehringer Mannheim). The cell culture was maintained at 37°C in a humidified atmosphere of 5% carbon dioxide in air.

At 8 days after transplantation, when the tumour diameter was about 0.6 cm, the B16 tumour-bearing mice were intravenously injected with a liposomally formulated BPD-MA (4 mg $kg^{-1}$ body weight) obtained from Quadra Logic Technologies (Vancouver, BC, Canada). Aliquots of BPD-MA were reconstituted from the lyophilized powder with distilled water at a concentration of 1.47 mg $ml^{-1}$ and stored, protected from light at 4°C. Further dilutions were carried out immediately before individual experiments using 5% dextrose in water.

At predetermined times after injection, the mice were sacrificed and the BPD-MA content in the serum and selected tissues was determined by spectrophotofluorimetric procedures. Briefly, the blood was centrifuged to remove the erythroctyes and appropriately diluted with 2% aqueous sodium dodecyl sulphate (SDS). The tissue specimens were homogenized in 2% SDS, the homogenate was tenfold diluted with a chloroform–methanol binary

*Received 11 March 1998*
*Revised 22 May 1998*
*Accepted 28 May 1998*

*Correspondence to*: A Busetti, Department of Biology, University of Padova, via U. Bassi 58/B, I-35121 Padova, Italy

*The contributions of the first two authors were equal.

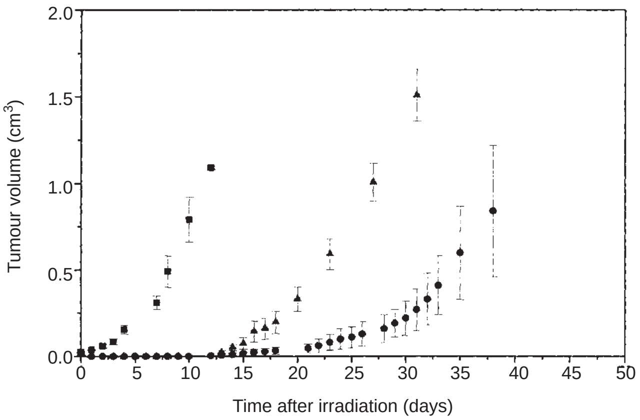

**Figure 1** Effect of 690-nm light irradiation (300 mW $cm^{-2}$) on the rate of tumour growth in C57/BL mice bearing a B16 pigmented melanoma, which had been i.v. injected with BPD-MA (4 mg $kg^{-1}$). Irradiations were performed at 3 h (●) and 9 h (▲) after injection. (■) Control unirradiated mice. Each point represents the average of seven mice ± s.d.

mixture (1:2, v/v) and centrifuged for 10 min at 3000 r.p.m.; the supernatant was collected and analysed for its BPD-MA content by fluorescence spectroscopy (excitation at 400 nm, emission at 650–760 nm), using a calibration plot built with known concentrations of BPD-MA in the same solvent mixture.

For the phototherapeutic studies, the tumour area was irradiated at 3 h or 9 h after i.v. administration of BPD-MA (4 mg $kg^{-1}$) using an argon-pumped dye laser with emission at 690 nm. The fluence rate ranged between 180 and 300 mW $cm^{-2}$ for a total light fluence of 300 or 520 J $cm^{-2}$. Under these irradiation conditions, the temperature of the tumour tissue increased to 38°C from a basal value of 33°C; this increase is below the extent required to give hyperthermal effects in the same animal model, as shown previously (Biolo et al, 1996). In a different set of experiments, the tumour area of the BPD-MA injected mice was irradiated with one pulse of 1064-nm light from a Q-switched Nd:YAG laser which was operated in a pulsed mode (10 ns pulses, 10 Hz, energy per pulse 650 mJ) and, immediately after, it was exposed to PDT treatment. In all cases, the effectiveness of the treatment was evaluated by comparing the rate of tumour growth of the irradiated mice with that observed for control mice transplanted simultaneously with the phototreated mice but not injected with BPD-MA and not exposed to light. The tumour size was measured daily by means of a calliper. Individual tumour volumes ($V$) were calculated by assuming a hemiellipsoidal structure for the tumour nodule and measuring the two perpendicular axes ($a$ and $b$) and the height ($c$). Application of the relationship $V = 2/3\pi(a/2 \times b/2 \times c)$ provided the tumour volume. The number of days for the tumour volume to reach 1 $cm^3$ was calculated for the individual tumours.

### RESULTS AND DISCUSSION

The pharmacokinetic and phototherapeutic studies with C57/BL6 mice bearing the B16 pigmented melanoma were performed by i.v. injection of 4 mg, i.e. 5.5 μmol of BPD-MA $kg^{-1}$ body weight. This dosage was chosen because previous reports (Allison et al, 1991) have shown that this amount of BPD-MA is effective in the PDT of other experimental tumours. Moreover, our initial investigations on the PDT of melanotic melanoma with SiNc (Biolo et al, 1996) were successfully carried out by administration of 0.64 μmol $kg^{-1}$ photosensitizer; as the extinction coefficient of SiNc and BPD-MA at the wavelengths used for their in vivo excitation are 557 000 $M^{-1}$ $cm^{-1}$ (773 nm) and 33 000 $M^{-1}$ $cm^{-1}$ (690 nm), respectively, the injection of an approximately 8.5-fold larger dose of BPD-MA should compensate, at least in principle, for the lower efficiency of light absorption. The recoveries of BPD-MA from the tumour, selected normal tissues and serum at different post-injection times are summarized in Table 1. The data represent the average recoveries from three independently analysed mice. An overall examination of the data suggests the following conclusions:

(1) BPD-MA is readily accumulated by the melanotic melanoma reaching maximum concentrations (1.8–1.9 nmol $g^{-1}$) at 1–3 h after i.v. administration. In the same animal model, SiNc yielded largest tumour concentrations of 0.33 nmol $g^{-1}$ at 24 h after injection, i.e. about 5.7-fold lower concentrations than those found for BPD-MA; hence, BPD-MA appears to be a slightly less efficient localizer of this specific tumour, because it had been injected in 8.5-fold larger concentrations (see above).

(2) The selectivity index of tumour targeting is given by the ratio between the photosensitizer concentration in the tumour to the peritumoral tissue, which is represented by the skin in our case. We analysed the BPD-MA recovery from skin areas adjacent and distal to the neoplastic lesion, as melanotic melanoma is known to undergo a radial diffusion, thereby readily infiltrating the surrounding cutaneous districts (Wosko et al, 1994). Our findings suggest that a limited selectivity of melanoma targeting by BPD-MA occurs only at 1–3 h with a concentration ratio of 1.6. In contrast, no selectivity of accumulation in the melanotic melanoma was observed for SiNc (Biolo et al, 1994; Biolo et al, 1996). Both BPD-MA and SiNc were found to

**Table 1** Recovery of BPD-MA from selected tissues (nmol $g^{-1}$) and serum (nmol $ml^{-1}$) of C57/BL6 mice bearing a B16 pigmented melanoma at various times after i.v. injection of the photosensitizer (5.5 μmol $kg^{-1}$).

| Time | Tumour | Peritumoral skin | Distal skin | Liver | Spleen | Serum |
|---|---|---|---|---|---|---|
| 30 min | 0.92 ± 0.23 | 0.79 ± 0.12 | 0.65 ± 0.14 | 44.53 ± 9.22 | 1.94 ± 0.18 | 9.30 ± 1.77 |
| 1 h | 1.94 ± 0.22 | 1.31 ± 0.42 | 0.90 ± 0.20 | 29.61 ± 9.34 | 1.38 ± 041 | 6.49 ± 0.94 |
| 3 h | 1.86 ± 0.10 | 1.13 ± 0.18 | 0.83 ± 0.57 | 15.64 ± 3.05 | 0.40 ± 0.11 | 4.40 ± 0.60 |
| 6 h | 1.05 ± 0.13 | 0.95 ± 0.22 | 0.72 ± 0.23 | 11.84 ± 1.44 | 0.25 ± 0.13 | 4.17 ± 0.86 |
| 9 h | 0.93 ± 0.01 | 0.71 ± 0.03 | 0.87 ± 0.17 | 6.15 ± 0.91 | nd | 3.23 ± 0.69 |
| 24 h | nd | nd | nd | 0.89 ± 0.16 | nd | 0.74 ± 0.06 |

nd, not detectable. Values ± s.d.; three mice were independently analysed at each time.

© Cancer Research Campaign 1999

give a good selectivity of tumour targeting in other transplanted tumours (Cuomo et al, 1990; Richter et al, 1990). The poor degree of selectivity observed in our experimental model is likely to reflect the fact that the skin of C57/BL6 mice accumulates unusually high amounts of photosensitizing agents.

(3) The large amount of BPD-MA recovered from liver suggests that this drug is mainly cleared from the organism via the bile–gut pathway, as it is typical of most liposome-delivered dyes (Jori, 1987).

(4) BPD-MA is eliminated at a remarkably fast rate, as at 24 h after injection residual small amounts of this porphyrin are still present only in the liver and serum. This property of BPD-MA is of particular importance for minimizing the occurrence of undesired side-effects, such as long-term liver toxicity or the persistence of generalized cutaneous photosensitivity for some weeks after the PDT treatment (Marcus, 1992). In this connection, BPD-MA is superior to SiNc, whose clearance from the organism is still incomplete 1 week after i.v. injection (Biolo et al, 1994).

On the basis of the pharmacokinetic studies, the PDT treatment of the B16 pigmented melanoma was performed at 3 h after the administration of 4 mg $kg^{-1}$ BPD-MA. When the irradiation protocol involved the delivery of an overall light dose of 520 mJ $cm^{-2}$, we observed a rapid development of an extensive necrotic area in the neoplastic mass as well as of peritumoral oedema; this was accompanied by a marked reduction in the rate of tumour growth as compared with that typical of control C57/BL6 mice (Figure 1). The PDT-treated mice appeared to be tumour free for almost 2 weeks. The delay of melanotic melanoma regrowth, as calculated from the data plotted in Figure 1, was 28 days (Table 2). No detectable tumour response was observed when the neoplastic lesion was exposed to 690-nm light under the same experimental conditions but in the absence of BPD-MA.

The extent of tumour response appeared to be correlated with the photosensitizer concentration in the malignant tissue: in fact, when the irradiation was performed under the same experimental conditions but at 9 h post injection, i.e. when the BPD-MA concentration in the tumour was decreased by about 50% as compared with that at 3 h, a significant response of the tumour was still observed; however, the regrowth delay was about 14 days (Figure 1). Similarly, a drop in the extent of tumour response to PDT took place upon reduction of the total light fluence and the fluence rate (Table 2). BPD-MA appears to be endowed with a high phototherapeutic activity towards the pigmented melanoma, as the PDT of B16 melanoma with SiNc gave a maximum delay of 4 days for tumour regrowth when applied using optimal light/photosensitizer doses (Biolo et al, 1996). The photoactivity of BPD-MA is similar to that observed for Lutexaphyrin, even though the latter porphyrin absorbs at longer wavelengths (Woodburn et al, 1997).

The effectiveness of BPD-MA was unexpected because the available data on the optical properties of heavily pigmented tissues, including melanotic melanoma, suggest that there is essentially no light transmittance up to about 720–730 nm. All the photosensitizers that so far have given positive results in the PDT of melanotic melanoma are characterized by the presence of intense absorption bands at wavelengths longer than 700 nm. It is possible that an initial photoinduced damage of superficial tissue layers is efficiently propagated throughout the tumour mass, which would be in agreement with the observation that BPD-MA predominantly acts by impairing the vascular system (Levy et al, 1994).

In all cases, pretreatment of the pigmented melanoma with high peak power 1064-nm laser radiation strongly enhanced the efficiency of the subsequent PDT treatment, as one can again deduce from the tumour regrowth delay data tabulated in Table 2. The 1064 + 690 nm irradiation studies were performed under suboptimal conditions, namely at 9 h post injection of BPD-MA. When the same studies were repeated at 3 h post injection, corresponding with maximal BPD-MA accumulation in the tumour, frequent death of phototreated mice was observed. Preliminary studies (Table 2; see also Busetti et al, 1998) showed that the Nd:YAG irradiation of B16 pigmented melanoma induces a specific breakdown of melanosomes with a modest (3–4 days) delay of tumour growth. Therefore, the observed enhancement of the tumour response to PDT with BPD-MA cannot be exclusively ascribed to the effect of the high peak power irradiation. Rather, such enhancement could be the result of the reduced optical filtering by the melanin pigments and the limited damage of the melanoma vascular system, i.e. the preferential site of the BPD-MA photosensitizing action. Similarly, the association of the SiNc-PDT with photoinduced thermal effects (Biolo et al, 1996) improved the response of B16 pigmented melanoma from a 4- to a 13-day regrowth delay.

**Table 2** Effect of different irradiation protocols performed 9 h post-injection of BPD-MA (5.5 μmol $kg^{-1}$) on regrowth of subcutaneously transplanted B16 pigmented melanoma in C57/BL6 mice (seven mice per group).

| Irradiation wavelengths (nm) | Irradiation parameters mJ/pulse + mW/$cm^{-2}$ –J/$cm^{-2}$ | Tumour free time (days) | Regrowth delay (days)[a] |
|---|---|---|---|
| 1064 | 650 | 0 | 3.7 ± 1.1 |
| 690[b] | 300–520 | 0 | 0.0 ± 0.2 |
| 690[c] | 300–520 | 15 | 28.0 ± 3.1 |
| 690 | 300–520 | 10 | 14.3 ± 1.0 |
| 1064 + 690 | 650 + 300–520 | 15 | 24.0 ± 0.9 |
| 690 | 300–300 | 0 | 5.0 ± 2.9 |
| 1064 + 690 | 650 + 300–300 | 7 | 19.5 ± 2.0 |
| 690 | 180–300 | 0 | 3.7 ± 2.2 |
| 1064 + 690 | 650 + 180–300 | 6 | 17.0 ± 0.6 |

[a]Difference between the growth time for treated and control mice. The growth time is the time interval for the tumour to grow to a volume of 1 $cm^3$ from the size at the time of irradiation (0.03–0.04 $cm^3$). The growth time of control mice was 11 days. [b]Control mice irradiated without injection of BPD-MA. [c]The irradiation was performed 3 h post BPD-MA injection.

© Cancer Research Campaign 1999

A more precise interpretation of the present findings will hopefully be provided by studies on the mechanism by which BPD-MA induces the necrosis of B16 melanotic melanoma, which are in progress in our laboratory. In any case, our findings clearly show that this porphyrin can also be successfully used in the treatment of pigmented tumours, thereby extending the scope of PDT. The efficacy of the phototherapeutic modality can be further enhanced by the application of photothermal treatment immediately before PDT.

### ACKNOWLEDGEMENTS

Partial support of this work came from NIH grant (CA46281) and from the Center for Photochemical Sciences of Bowling Green State University. The authors wish to acknowledge the valuable assistance of Svetlana Leytner, Center for Photochemical Sciences, Bowling Green State University, and QLT PhotoTherapeutics Inc. for the gift of BPD-MA.

### REFERENCES

Allison BA, Waterfield E, Richter AM and Levy JG (1991) The effects of plasma lipoproteins on in vitro tumor cell killing and in vivo tumor photosensitization with benzoporphyrin derivative. *Photochem Photobiol* **54**: 709–715

Biolo R, Jori G, Soncin M, Pratesi R, Vanni U, Rihter B, Kenney ME and Rodgers MAJ (1994) Photodynamic therapy of B16 pigmented melanoma with liposome-delivered Si(IV)-naphthalocyanine. *Photochem Photobiol* **59**: 362–365

Biolo R, Jori G, Soncin M, Rihter M, Kenney ME and Rodgers MAJ (1996) Effect of photosensitizer delivery system and irradiation parameters on the efficiency of photodynamic therapy of B16 pigmented melanoma. *Photochem Photobiol* **63**: 224–228

Busetti A, Soncin M, Jori G, Kenney ME and Rodgers MAJ (1998) Treatment of cutaneous malignant melanoma by high peak power 1,064 nm irradiation followed by photodynamic therapy. *Photochem Photobiol* **8**: 377–381

Cuomo V, Jori G, Rihter B, Kenney ME and Rodgers MAJ (1990) Liposome-delivered Si(IV)-naphthalocyanine as a photodynamic sensitizer for experimental tumours: pharmacokinetic and phototherapeutic studies. *Br J Cancer* **62**: 966–970

Dougherty TJ (1987) Photosensitizers: therapy and detection of malignant tumours. *Photochem Photobiol* **45**: 879–889

Jori G (1987) Photodynamic therapy of solid tumours. *Radiat Phys Chem* **30**: 375–380

Levy S (1996) New applications in photodynamic therapy. *Photochem Photobiol* **64**: 737–739

Levy JG, Waterfield E, Richter AM, Smits C, Lui H, Hruza L and Anderson RR (1994) Photodynamic therapy of malignancies with benzoporphyrin derivative monoacid ring A. In *Photodynamic Therapy of Cancer*, Vol. 2,074. Jori G, Moan J and Star WN (eds), pp. 91–101. SPIE: Bellingham, WA

Marcus S (1992) Clinical photodynamic therapy: the continuing evolution. In *Photodynamic Therapy*: *Basic Principles and Clinical Application*. Henderson BW and Dougherty TJ (eds), pp. 219–268. Marcel Dekker: New York

Richter AM, Cerruti-Sola S, Sternberg ED, Dolphin D and Levy JG (1990) Biodistribution of tritiated benzoporphyrin derivative, a new potent photosensitizer in normal and tumour-bearing mice. *J Photochem Photobiol B*: *Biol* **5**: 231–244

Schuitmaker JJ, van Best JA, van Delft JL, Jannink JE, Oosterhuis JA, Vrensen GFJM, de Wolff-Rouendaal D and Dubbelman TMAR (1995) Photodynamic therapy of hamster greene melanoma in vitro and in vivo using Bacteriochlorin a as photosensitizer. In *Photochemotherapy*: *Photodynamic Therapy and Other Modalities*, Vol. 2,625. Ehrenberg B, Jori G and Moan J (eds), pp. 251–260. SPIE: Bellingham, WA

Svaasand LO, Martinelli E, Gomer CJ Profio AE (1990) Optical characteristics of intraocular tumours in the visible and near infrared. In *Photodynamic Therapy*: *Mechanisms II*, Vol. 1,203. Dougherty TJ and Katzir A (eds), pp. 2–21. SPIE: Bellingham, WA

Woodburn W, Qing F, Kessel D, Luo Y and Young W (1997) Photodynamic therapy of pigmented melanoma with Lutetium Texaphyrin. *Photochem Photobiol* **65**: 59S

Wosko TJ, Ferrara DT and Sartori TS (1994) Histological comparison of the B16 melanoma and its F1 variant. *Cancer Lett* **24**: 57–63

 *© Cancer Research Campaign 1999*
